# Development of a tropoelastin-binding MR contrast agent for *in vivo* imaging of impaired elastogenesis in atherosclerosis

**DOI:** 10.1186/1532-429X-17-S1-O102

**Published:** 2015-02-03

**Authors:** Alkystis Phinikaridou, Sara Lacerda, Marcelo E Andia, Rene Botnar

**Affiliations:** 1 Biomedical Egineering, King's College London, London, UK; 2 Radiology, Pontificia Universidad Católica de Chile, Santiago, Chile

## Background

Elastin is a primary component of the vessel wall and present in all elastic vessels such as the aorta and the pulmonary artery. Elastogenesis begins with the synthesis and secretion of the soluble precursor tropoelastin that becomes cross-linked into insoluble elastin. Under normal conditions cross-linked elastin is the only form of the molecule present in the vessel wall whereas tropoelastin is absent. Conversely, under pathological conditions including atherosclerosis and aortic aneurysms elastogenesis resumes and tropoelastin molecules accumulate in the vessel wall. We developed a novel tropoelastin-binding MRI contrast-agent that would allow 1) specifically bind to tropoelastin but not to cross-linked mature elastin to allow 2) detection of pathologic elastogenesis that occurs in atherosclerosis and 3) minimizing non-specific signal from endogenously present mature elastin.

## Methods

Two tropoelastin-binding peptides (Gd-DOTA)-*VVGSPSAQDEASPLS* and K(Gd-DOTA)*YPDHVQYTHY* were tested. *In vitro* binding studies were performed using Europium-labeled peptides and a DELFIA method. *In vivo* MRI of the aortic and brachiocephalic arteries was performed in atherosclerotic ApoE^-/-^ and control mice using a 3T Philips Achieva scanner and a single loop microscopy surface coil. Images were acquired for up to 1h after intravenous administration of 0.2 mmol/kg tropoelastin-binding probes. 3D gradient-echo DE-MRI images were acquired with FOV=30x8x30mm, matrix=300, resolution=0.1x0.1mm, slice thickness=0.25mm, TR/TE=27/8ms, TR between subsequent IR pulses=1000ms, and flip angle=30°. T1 mapping was performed using a 3D modified Lock-Locker sequence FOV=22x8x36, matrix=180x171, resolution=0.2x0.2, slice thickness=0.5mm, TR/TE= 9.2/4.7ms, flip angle=10°. T1 values were computed on a pixel-by-pixel basis using an in-house Matlab algorithm.

## Results

**Figure 1:**
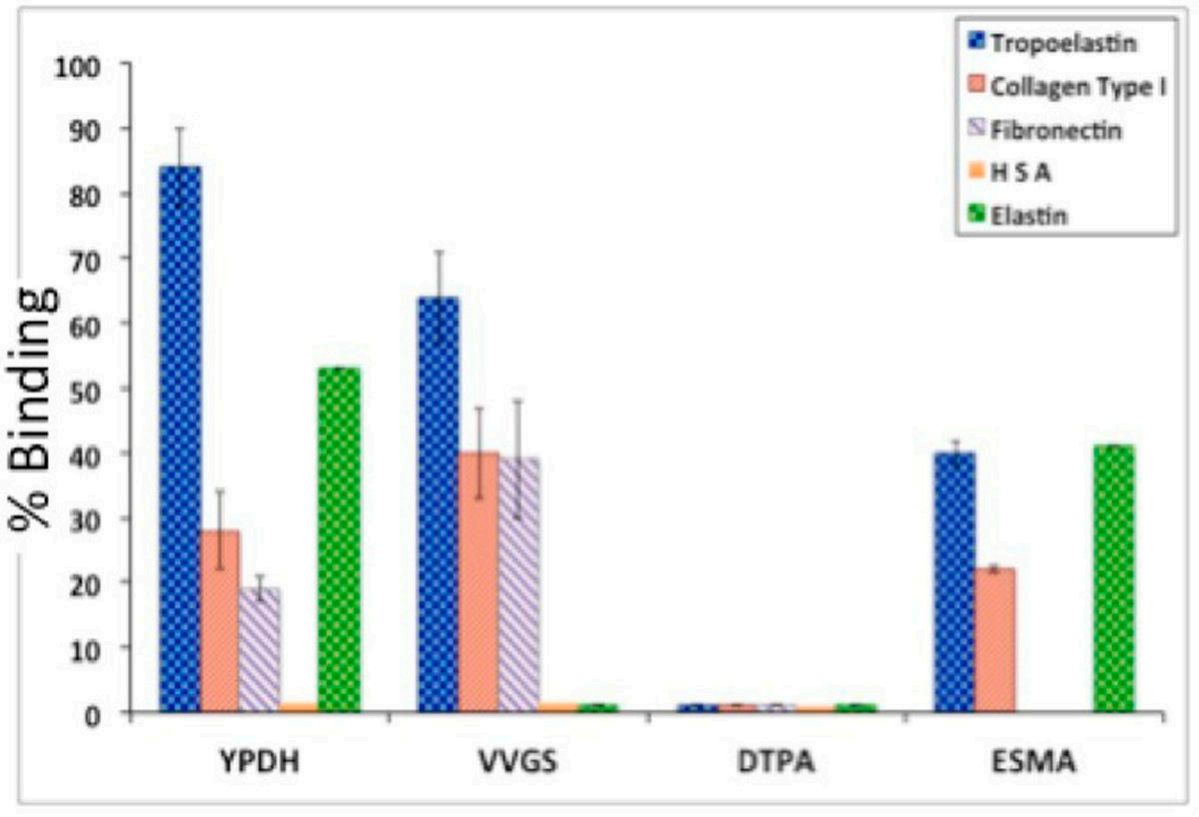
In vitro binding assays.

**Figure 2:**
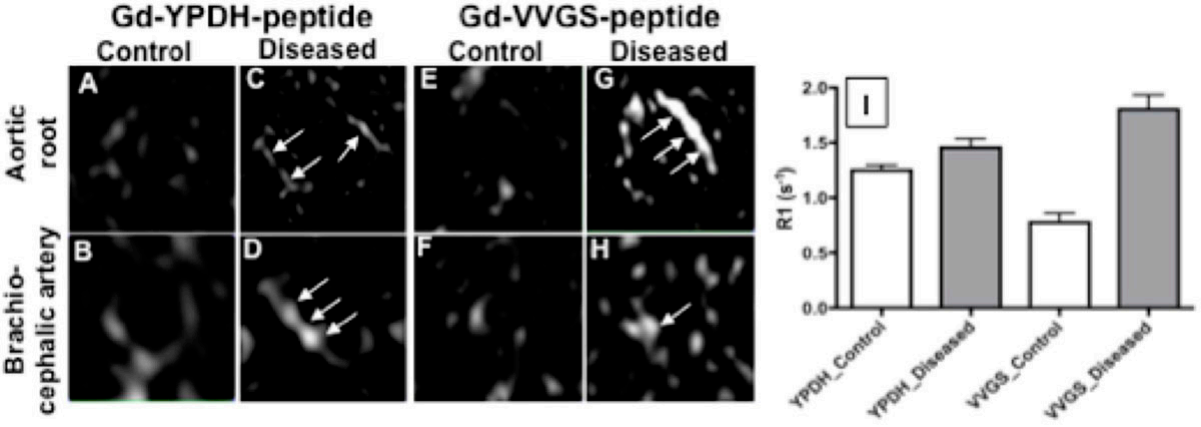
*In vivo* MRI experiments using the YPDH and VVGS peptides in control and atherosclerotic mice.

*In vitro* binding assays showed high selectivity of the compounds towards tropoelastin compared to other proteins and particularly mature elastin (Fig 1). The VVGS probe achieved best discrimination between tropoelastin and mature elastin. Similarly, DE-MRI *in vivo* images showed enhancement of the diseased vessel wall (Fig. 2C-D, G-H; arrows), where tropoelastin is present, and less or no uptake in control animals (Fig. 2A-2B, E-F) where tropoelastin is absent. Quantitative analysis of the vessel wall R1 showed a greater difference in R1 between control and disease vessels walls using the VVGS based probe (Fig. 2I).

## Conclusions

We developed of a new gadolinium-based tropoelastin-binding contrast agent for imaging atherosclerosis. Both peptide-based probes showed promising pharmacokinetics, specificity and sensitivity for *in vivo* MRI imaging diseased brachiocephalic arteries in ApoE^-/-^ mice. Further development of such contrast agent may allow for molecular imaging of impaired elastogenesis that accompany atherosclerotic plaque development and plaque vulnerability.

## Funding

British Heart Foundation (RG/12/1/29262).

